# O.6.1-2 Motor competencies of elementary-school aged children – investigating the conjoint impact of parenting, school grounds quality, children’s movement behavior and self-confidence

**DOI:** 10.1093/eurpub/ckad133.261

**Published:** 2023-09-11

**Authors:** Beatrix Algurén, Yiling Tang, Chelsea Pelletier, P-J Naylor, Guy Faulkner

**Affiliations:** University of Gothenburg, Sweden; University of British Columbia, Canada; beatrix.alguren@gu.se; University of British Columbia, Canada; beatrix.alguren@gu.se; University of British Columbia, Canada; beatrix.alguren@gu.se; University of British Columbia, Canada; beatrix.alguren@gu.se

## Abstract

**Purpose:**

To understand factors that impact on children’s motor competencies (MCs) from a socio-ecological perspective.

**Methods:**

The study is a cross-sectional sub-study of a three-year longitudinal cohort study called ‘Physical Literacy for communities (PL4C)’ in the West Vancouver school district. Motor competencies were assessed by the Physical Literacy Assessment for Youth (PLAY) Fun tool. Children were asked to perform 18 different movement tasks across five domains: running, locomotor, upper and lower body control, balance.

Children’s objective physical activity was measured using wrist-worn accelerometer Actigraph GT3X+BT. Through a survey, parents were asked about their children’s amount of time playing outside during workdays and weekdays as well as time doing sports or instructor-led physical activity.

The Activity Support Scale for Multiple Groups (ACTS-MG) were included in the survey measuring parental support for children’s physical activity.

Quality of the 14 school grounds was measured by using the Sport, Physical activity and Eating behavior: Environmental Determinants in young people (SPEEDY) school grounds audit tool existing of six component scores: cycling provision, walking provision, sports and play facilities provision, other facility provision, design of the school grounds, aesthetics.

**Results:**

Complete assessment of motor competencies (with PlayFun) were done for 319 children with average age of 7.5 years (90%; 166 boys, 147 girls, 6 non-binaries), whereof the majority was on an emerging- level (79%) regarding overall MC and one fifth on competent- level (n = 67). Participants accumulated MVPA about 111 minutes per day (113 min/d for boys, 109 min/d for girls, p = 0.37). Children with competent-level MC had in average 15 minutes/day more MVPA compared to children with emerging-level MC (p = 0.001). Analysis with structural equation modeling (SEM) revealed that direct significant effects of parenting, school grounds quality, children’s movement behavior varied for each MC and overall MC and between gender. ‘Logistics’ turned out to influence significantly positive most often, and had a positive significant effect on males and females overall MC (g = 0.267, p = 0.004 and g = 0.247, p = 0.011).

**Conclusions:**

Beside parents logistics support and participation in sports, school grounds seems to impact positively children’s MCs, yet different school grounds qualities enhance girls’ and boys’ MCs in different ways.

